# Regulatory mechanism of a heat-activated retrotransposon by DDR complex in *Arabidopsis thaliana*

**DOI:** 10.3389/fpls.2022.1048957

**Published:** 2022-12-21

**Authors:** Xiaoying Niu, Lu Chen, Atsushi Kato, Hidetaka Ito

**Affiliations:** ^1^ Graduate School of Life Science, Hokkaido University, Sapporo, Hokkaido, Japan; ^2^ Faculty of Science, Hokkaido University, Sapporo, Hokkaido, Japan

**Keywords:** epigenetics, transposons, environmental stress, *Arabidopsis thaliana*, *ONSEN*

## Abstract

The RNA-directed DNA methylation (RdDM) pathway plays an essential role in the transposon silencing mechanism; the DDR complex, consisting of DRD1, DMS3, and RDM1, is an essential component of the RdDM pathway. *ONSEN*, identified in *Arabidopsis*, is a retrotransposon activated by heat stress at 37°C; however, studies on the regulation of *ONSEN* are limited. In this study, we analyzed the regulation of *ONSEN* activity by the DDR complex in *Arabidopsis*. We elucidated that loss of any component of the DDR complex increased *ONSEN* transcript levels. Transgenerational transposition of *ONSEN* was observed in the DDR-complex mutants treated with heat stress for 48 h. Furthermore, the DDR complex components DRD1, DMS3, and RDM1 played independent roles in suppressing *ONSEN* transcription and transposition. Moreover, we found that the duration of heat stress affects *ONSEN* activity. Therefore, the results of this study provide new insights into the retrotransposon regulatory mechanisms of the DDR complex in the RdDM pathway.

## 1 Introduction

Transposable elements (TEs) are DNA sequences that move around in the host genome; they are widely found in most eukaryotic genomes. TEs are found in 60–80%, 29%, and 17% of maize, rice, and *Arabidopsis* genomes, respectively (Le et al., 2000; Bennetzen, 2005). TEs are classified into two major groups based on their transposition mechanism: type I transposons (retrotransposons) and type II transposons (DNA transposons) (Wessler, 2006). Retrotransposons are the most common type of TEs and are a major component of plant genomes. Retrotransposons use the “copy-and-paste” mechanism for transposition. They can be divided into two main groups: the long terminal repeat (LTR) group and the non-LTR group, based on whether the end of the element has an LTR sequence (Wicker et al., 2007).

When plants are exposed to the external environment, especially to adverse conditions such as high temperature, salt stress, low temperature, etc., the retrotransposons are activated; they insert into the target genes and simultaneously affect the expression of the surrounding genes (Lisch, 2013; Ito, 2022; Thieme et al., 2022). Since the transcription of retrotransposons affects the stability of plant genomes, almost all transposons are silenced by epigenetic modifications, such as DNA methylation and histone modifications (Zilberman et al., 2007; Zhang et al., 2010). DNA methylation can affect gene expression; the changes can be passed on to the next generations *via* cell division without any changes in the DNA sequence (Bird, 2007; Heard and Martienssen, 2014). DNA methylation patterns are regulated by the small interfering RNA (siRNA) pathway (Pontes et al., 2006). The siRNA pathway-directed DNA methylation pattern leads to transcriptional gene silencing (TGS) at the transcriptional level (Wakasa et al., 2018). The RNA-directed DNA methylation (RdDM) pathway is broadly classified into canonical and non-canonical RdDM pathways. The most studied canonical RdDM depends on two plant-specific RNA polymerases, RNA polymerase IV (Pol IV) and RNA polymerase V (Pol V) (Zhong et al., 2012; Matzke and Mosher, 2014; Xue et al., 2021).

In *Arabidopsis*, Pol IV is involved in the production of siRNA, while Pol V is required to recruit DRM2 methyltransferase to establish the methylation pattern in the siRNA-complementary genome sequences. (Matzke et al., 2009). The DDR (DRD1-DMS3-RDM1) complex is required for Pol V-dependent transcription, acting upstream of Pol V to regulate Pol V activity and stabilize Pol V binding to chromatin (Law et al., 2010). The DDR complex consists of the following proteins: DEFECTIVE IN RNA-DIRECTED DNA METHYLATION 1 (DRD1), DEFECTIVE IN MERISTEM SILENCING 3 (DMS3), and RNA-DIRECTED DNA METHYLATION 1 (RDM1); impaired function of any of these proteins results in impaired RdDM activity (Haag and Pikaard, 2011; Zhong et al., 2012; Zhong et al., 2018). DRD1 is a plant-specific SNF2-like putative chromatin remodeling protein (Kanno et al., 2004; Kanno et al., 2005), which assists in the physical association of Pol V with intergenic loci and generates transcripts that repress adjacent transposons by establishing repressive heterochromatin (Wierzbicki et al., 2008). DMS3 structure is similar to the hinge domain region maintained by chromosomal (SMC) protein, which may be involved in secondary siRNA production. RDM1 binds to single-stranded methyl DNA, which may attach to single-stranded DNA at the site of Pol V transcription, and thus, contribute to the recruitment of Pol V to RdDM targets in the perinuclear processing centers (Gao et al., 2010). DMS3 and RDM1 form a structurally stable DR complex. Moreover, the interaction between DMS3 and RDM1 is a prerequisite for forming a stable complex between DR and DRD1 (Wongpalee et al., 2019).

Many recent reports have demonstrated that environmental stress can activate TEs (Ito, 2022; Steward et al., 2002). *ONSEN* is a heat-activated retrotransposon identified in *Arabidopsis* (Ito et al., 2011). After heat-stress (HS) at 37°C, *ONSEN* is transcriptionally active and synthesizes extrachromosomal DNA. The transgenerational transposition of *ONSEN* has not yet been confirmed in the wild-type plant. *ONSEN* exhibits high transcript levels in *nrpd1* mutant plants (a loss-of-function mutant of POL IV), and the new transposition occurs in the next generation. However, little information is available regarding the transpositional regulation of *ONSEN* (Ito et al., 2011; Matsunaga et al., 2015).

This study investigated *ONSEN* transcription and transposition in single and multiple mutants associated with the DDR complex. We found that HS activates the transcription of *ONSEN* in DDR complex-associated mutants. The transposition of *ONSEN* occurred in *ddr* mutant after HS (37°C) for 48 h. Moreover, we found that the duration of HS influences the effect of RdDM pathway factors on *ONSEN* silencing. This study reveals the repression of *ONSEN* by the DDR complex in the RdDM pathway. It also elucidates the independent role of RdDM factors in the mechanism behind the repression of transcriptional and transpositional activity of retrotransposons.

## 2 Materials and methods

### 2.1 Plant material

*Arabidopsis thaliana* accession Columbia (Col-0) was used to develop all mutants. The *drd1* (CS69758), *dms3* (CS69174), *rdm1* (CS69759), and *nrpd1* (C366150) mutants were obtained from Ohio State University, Columbus, OH, USA; *drd1* mutant was crossed with *dms3* or *rdm1* mutant to generate *drd1/dms3* and *drd1/rdm1* mutants, and *drd1/dms3* mutant was crossed with *drd1/rdm1* mutant to generate the *drd1/dms3/rdm1* (*ddr*) mutant.

### 2.2 Growth conditions

The plants were grown on 0.5× Murashige and Skoog (MS) basal medium or potting medium (soil: vermiculite = 1:7) in continuous light at 21°C after 2 d of cold treatment at 4°C (dark condition). The seeds were sterilized in a 1:1 solution of sodium hypochlorite solution and 0.04% Triton X-100 before being sowed in the MS medium.

### 2.3 Heat-stress treatment

Seven-day-old seedlings grown under non-stress condition (continuous light at 21°C) were subjected to HS treatment at 37°C for 24 or 48 h. After the HS treatment, the seedlings were directly sampled for further analysis. For Southern blotting, the treated seedlings were grown under 21°C, and after a few days of recovery, the seedlings were potted in potting media.

### 2.4 DNA methylation analysis

The bisulfite whole genome sequencing data (GSE39901) for *drd1*, *dms3*, *rdm1*, *nrpd1*, and wild type were obtained from NCBI GEO. The data were imported into IGV (2.12.3) for data visualization after format conversion.

For Chop-PCR analysis, DNA from 7-day-old seedlings was extracted using the Nucleon PhytoPure DNA extraction kit (Cytiva Tokyo, Japan). We used 500 ng DNA for methylation-sensitive restriction enzyme (*Alu* I) digestion for 12 h at 37°C. PCR amplification (30 cycles of 94°C for 30 s, 55°C for 30 s, and 72°C for 1 min) was performed with the appropriate primers (**Supplementary Table 1**). The PCR products were separated into 0.8% agarose gels.

For Bisulfite Sanger sequencing, genomic DNA was isolated from 7-day-old seedlings using the Nucleon PhytoPure DNA extraction kit (Cytiva Tokyo, Japan). MethylCode bisulfite conversion kit (Invitrogen, Waltham, MA, USA; MECOV-50) was used for bisulfite conversion and desulfurization. After PCR amplification (40 cycles of 98°C for 10 s, 55°C for 30 s, and 72°C for 1.5 min) with the corresponding primers, the PCR products were transformed into *Escherichia coli* and then were cloned. Twenty-four clones of each corresponding region were sequenced. The sequencing results were compared to the original sequences using MEGA-X software. The comparison files were then uploaded to the Cymate website to analyze different background DNA methylation levels at each locus.

### 2.5 Quantitative analysis

For quantitative analysis, quantitative PCR (qPCR) was performed. DNA was extracted using the Nucleon phytoPure DNA extraction kit (Cytiva Tokyo, Japan). For quantitative reverse transcription PCR (RT-qPCR), RNA was extracted by TRI Reagent (Sigma-Aldrich, St. Louis, MI, USA; T9424). The extracted total RNA was treated with RQ1 RNase-Free DNase (Promega, Madison, WI, USA; M6101), and complementary DNA (cDNA) was synthesized from the treated RNA by reverse transcription using the ReverTra Ace^®^ qPCR RT Kit (TOYOBO, Osaka, Japan).

qPCR was performed on an Applied Biosystems 7300 Real-Time PCR System using Luna Universal qPCR Master Mix (New England BioLabs, Ipswich, MA, USA). The expression was determined by the Ct method, and the expression of the 18S rRNA gene was used as the internal control.

### 2.6 Southern blotting

DNA from 21-day-old whole plant tissue was extracted using the Nucleon PhytoPure DNA extraction kit (Cytiva Tokyo, Japan). The extracted DNA was treated overnight with the restriction enzyme *EcoR*V at 37°C. Next, the DNA was purified by ethanol-precipitation, and electrophoresis was performed on a 1% agarose gel at 20 V for 24 h; the DNA was transferred onto a membrane overnight. Hybridization signals were detected by the Megaprime DNA Labeling System (Cytiva Tokyo, Japan) using probes labeled with radioisotopes.

### 2.7 Cytology

Seven-day-old leaves were fixed with ethanol:acetic acid (3:1). The leaves were incubated at 37°C with cellulase (Yakult Pharmaceutical Industry Co., Ltd, Japan; Cellulase Onozuka R-10), pectinase (Kyowa Chemical Products, Tokyo, Japan; YAKPECT23-5), and cytohelicase (Sigma-Aldrich, St. Louis, MI, USA; C8274) at 1% (w/v) in citrate buffer for 2.5–3 h. Next, 45% acetic acid was added to the leaves, and they were placed on slides, which were placed on a heating plate at 45°C as the leaves were being choped with a needle; this was followed by the addition of a fixative. After drying the slides at 24°C, 4′,6-diamidino-2-phenylindole (DAPI) (Vector Laboratories, Inc. Newark, CA, USA; H-1200-10) was added to stain the leaf cells. Three hundred images were taken of each mutant grown under different conditions. The cells in the images captured by a fluorescence microscope were then automatically classified into three states: condensed, half dispersed, and dispersed, using machine learning that includes Create ML, RectLabel, and CutSort.

## 3 Results

### 3.1 Defective DDR complexes reduce DNA methylation of *ONSEN*

We investigated the genome-wide DNA methylation levels of *drd1*, *dms3*, *rdm1*, *nrpd1*, and the wild type using publicly available bs-seq data (**Supplementary Figure 1**). We found that the methylation levels of non-CG (CHG and CHH) were reduced in the *drd1*, *rdm1*, and *nrpd1* compared to those in the wild type, whereas the non-CG methylation levels were only slightly reduced in the *dms3* mutant. Moreover, there was no difference in CG methylation levels among all mutants.

**Figure 1:**
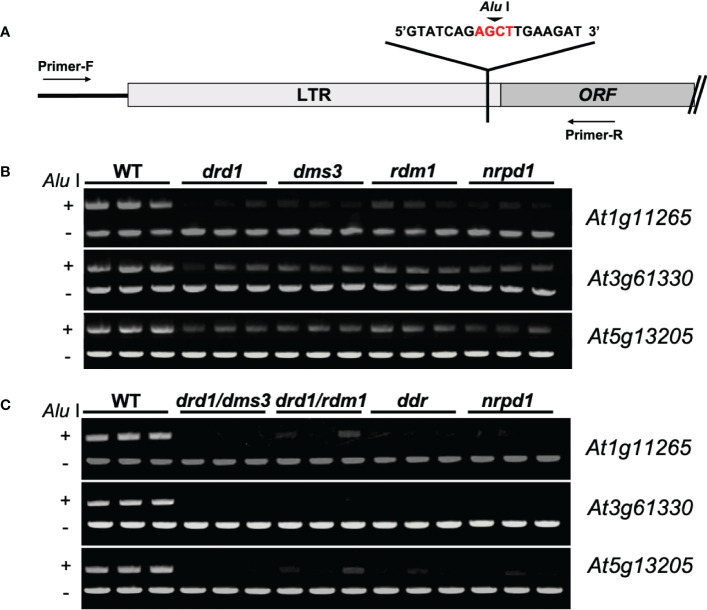
*ddr* deletion disrupts DNA methylation. **(A)** Primer sites for Chop-PCR amplification. Primers were designed to increase sequences in and around the *ONSEN* LTR region. AGCT is a sequence recognized by *Alu* I, which cuts the connection between G and C when C is not methylated. **(B)** *ONSEN* (*At1g11265*, *At3g61330*, and *At5g13205*) methylation levels in the wild type and *drd1*, *dms3*, *rdm1*, and *nrpd1* mutants. **(C)** *ONSEN* (*At1g11265*, *A3g61330*, and *At5g13205*) methylation levels in WT, *drd1/dms3*, *drd1/rdm1*, *ddr* and *nrpd1* mutants.

Previous studies have shown that eight copies of *ONSEN* are present in *Arabidopsis*, but the activity of each copy is different. Among them, three copies, i.e., *At1g11265*, *At3g61330*, and *At5g61305*, are the most active (Cavrak et al., 2014). Therefore, to investigate whether the methylation levels of *ONSEN* are different among *ONSEN* copies, we investigated the three copies *At1g11265*, *At3g61330*, and *At5g61305* in *drd1*, *dms3*, *rdm1*, *nrpd1*, and the wild type using a Chop-PCR (**Figures 1A–C**). The result showed that the methylation levels of all three copies were differentially reduced in the *drd1*, *dms3*, *rdm1*, and *nrpd1* mutants compared to those in the wild type (**Figure 1B**). This suggests that the deletion of *drd1*, *dms3*, and *rdm1* impairs *ONSEN* methylation. Next, to investigate the methylation levels of compound DDR mutants, we investigated the methylation levels of *ONSEN* in the *drd1/dms3*, *drd1/rdm1*, and *ddr* triple mutants by the Chop-PCR. The results revealed that simultaneous deletion of *drd1*, *dms3*, and *rdm1* reduced DNA methylation in *ONSEN* (**Figure 1C**). These data suggest that the DDR complex is required to maintain methylation on the *ONSEN* sequence.

### 3.2 Duration of heat stress affects the activity of *ONSEN*

**Figure 2:**
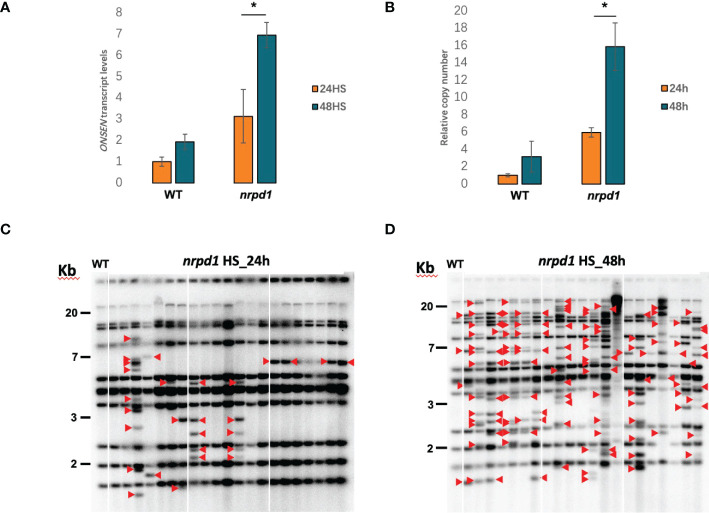
*ONSEN* activation affected by heat stress duration. **(A, B)** The relative expression levels **(A)** and relative copy numbers **(B)** of wild type and *nrpd1* mutant detected by RT-qPCR and qPCR under heat-stress (HS) conditions at 24 and 48 h for *ONSEN*. Asterisks indicate significant differences between the 24 and 48 h HS conditions (Student’s *t*-test, P < 0.05). **(C, D)** Southern blotting analysis of *ONSEN* in the next-generation *nrpd1* mutant under HS [24 h **(C)** and 48 h **(D)**]. Seven individuals of the next generation from each of the three parent plants subjected to heat stress were analyzed. The leftmost panel in each figure shows the wild-type pattern under non-stress (NS) condition. Red arrows indicate new *ONSEN* insertions.

*ONSEN* was expressed under HS conditions at 37°C. To investigate whether the duration of heat affects *ONSEN* activity, we examined the transcript levels of *ONSEN* in the wild type and *nrpd1* mutant under 24 and 48 h of HS by RT-qPCR. We found that *ONSEN* transcript levels were higher in plants subjected to 48 h of HS than in those subjected to 24 h of HS; the levels were significantly (P < 0.05) different in the *nrpd1* mutant compared to those in the wild type (**Figure 2A**). The transposition of retrotransposons requires three steps: transcription, cDNA synthesis, and insertion into other locations on the chromosome. To determine whether the copy number of *ONSEN* changes, we examined the copy number of *ONSEN* in the wild type and *nrpd1* mutants after 24 and 48 h of HS by qPCR. The results revealed that the copy number of *ONSEN* in the wild type and *nrpd1* mutant increased under 48 h of HS (**Figure 2B**). Next, we investigated the transposition of *ONSEN* under 24 and 48 h of HS using Southern blotting. The results showed that *ONSEN* transposition occurred in the *nrpd1* mutant after both 24 and 48 h of HS (**Figures 2C, D**). Moreover, *ONSEN* exhibited a higher transposition frequency in the *nrpd1* mutant after 48 h of HS (**Figure 2D**). However, the wild type did not exhibit transposition of *ONSEN* under the same conditions (**Supplementary Figure 2**). These results suggest that the duration of HS affects the activity of *ONSEN*, including the transcript level, copy number, and transposition frequency.

### 3.3 *ONSEN* was upregulated in DDR mutants under HS

**Figure 3:**
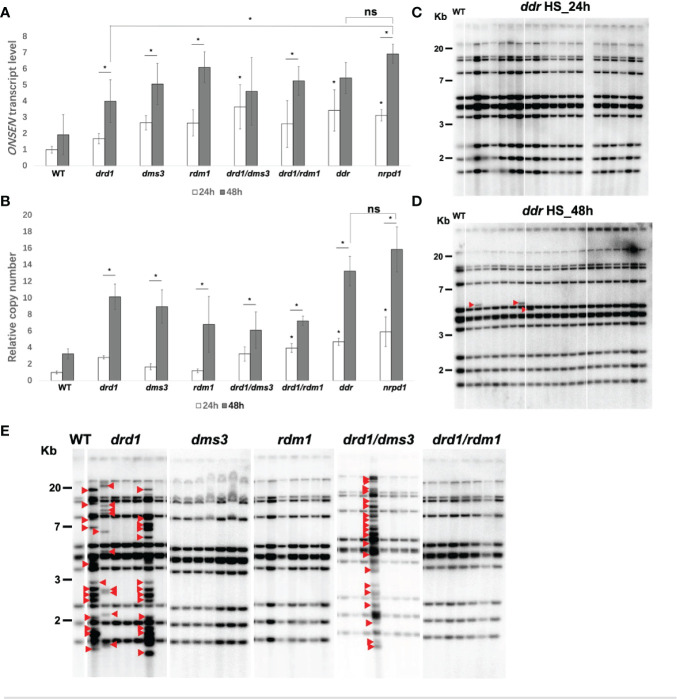
*ONSEN* activation in DDR complex-associated mutants subjected to heat stress (HS). **(A, B)** Comparative analysis of the expression level **(A)** and copy number **(B)** of *ONSEN* after 24 and 48 h of HS in wild type, *nrpd1*, and the DDR complex-associated mutants (Student’s t-test, P < 0.05). Asterisks indicate significant differences between the 24 and 48 h HS conditions (Student’s t-test, P < 0.05). ns indicates not significant. **(C, D)** Detection of transposition of *ONSEN* in the next generation (F2) plants of the *ddr* mutant after 24 h **(C)** and 48 h **(D)** HS. Seven individuals of the next generation from each of the three parent plants subjected to heat stress were analyzed by Southern blotting. Red arrows indicate new *ONSEN* insertions. **(E)** Southern blot analysis of *ONSEN* in the next generation plants of *drd1, dms3, rdm1, drd1/dms3*, and *drd1/rdm1* mutants after 48 h of HS. Seven individuals of the next generation from each of the parent plants subjected to heat stress were analyzed. Red arrows indicate new *ONSEN* insertions.

To investigate the role of the DDR complex in *ONSEN* transcription, we examined the transcript level of *ONSEN* in single and compound mutants of the DDR complex under HS conditions. We observed a significant increase in the *ONSEN* transcript levels in the *drd1/dms3*, *drd1/dms3/rdm1*, and *nrpd1* mutants under 24 h of HS compared to those of the wild type (**Figure 3A**). In addition, *ONSEN* expression levels were higher in the *drd1*, *dms3*, *rdm1*, *drd1/rdm1*, and *nrpd1* mutants after 48 h of HS than those after 24 h (**Figure 3A**). Thus, *ONSEN* transcription is activated in the DDR complex mutants under HS, suggesting that the DDR complex contributes to the transcriptional repression of *ONSEN* in the RdDM pathway. Moreover, the expression level of *ONSEN* increased with the duration of heat administration in each mutant. A significant difference in the transcription level was observed only in the *drd1* mutant compared to that in the *nrpd1* mutant after 48 h of HS (**Figure 3A**). *ONSEN* was not activated in the DDR complex-related mutants without heat stress (**Supplementary Figure 3A**).

Next, we investigated the copy number of *ONSEN* in single and compound DDR complex mutants after 24 and 48 h of HS. After 24 h of HS, *ONSEN* copy number was significantly increased in the *drd1/rdm1*, *drd1/dms3/rdm1*, and *nrpd1* mutants compared to that of the wild type (**Figure 3B**). Similar to the results obtained for transcript levels, *ONSEN* copy number increased in each mutant after 48 h of HS compared to 24 h (**Figure 3B**). These results indicate that the copy number of *ONSEN* increases in the DDR mutant plants under HS conditions, and the copy number is directly correlated with the duration of HS. In contrast, no change was observed in the copy number of *ONSEN* in each mutant under NS conditions (**Supplementary Figure 3B**).

### 3.4 DDR complex suppresses *ONSEN* transposition *via* DRD1

To investigate the role of the DDR complex in the inhibition of *ONSEN* transposition, the *ddr* triple mutants were subjected to HS for 24 and 48 h. Southern blotting was used to determine whether *ONSEN* transposition occurred in the progeny of the mutants. Although no *ONSEN* transposition was observed after 24 h of HS (**Figure 3C**), *ONSEN* transposition was observed after 48 h of HS (**Figure 3D**). The results suggest that the loss of function of the DDR complex leads to a release of *ONSEN* transposition under an extended period of HS. Next, we analyzed the transposition of *ONSEN* in the *drd1*, *dms3*, *rdm1*, *drd1/dms3*, and *drd1/rdm1* mutants subjected to 24 and 48 h of HS. The result showed that new insertions of *ONSEN* were observed in the *drd1* and *drd1/dms3* mutants after HS of 48 h (**Figure 3E**). However, *ONSEN* transposition was not observed in any of the mutants subjected to 24 h of HS (**Supplementary Figure 4**). Thus, the DDR complex suppresses *ONSEN* transposition, mainly through the role of DRD1.

### 3.5 Relaxation of heterochromatin by HS affects *ONSEN* transcriptional activity, but it does not correlate with the transposition frequency

**Figure 4:**
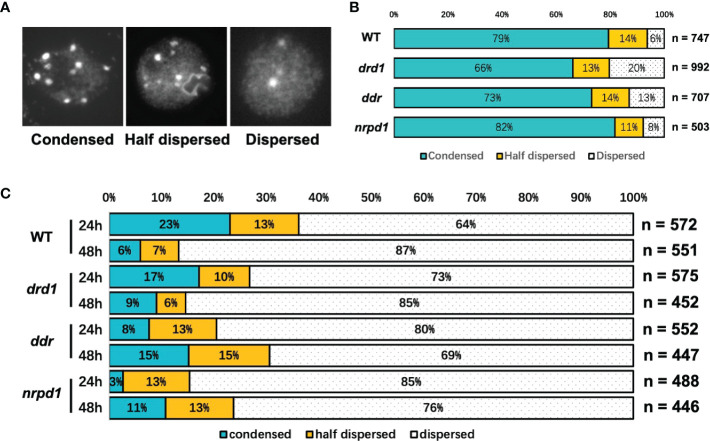
Heterochromatic condensation levels in DDR complex-associated mutants as per nuclear staining analysis. **(A)** Representative images of three heterochromatin states in the DAPI-stained nucleus include: condensed, half dispersed, and dispersed. **(B)** The bars show the percentage of the nuclei in the three states **(A)** in wild type and *drd1*, *ddr*, and *nrpd1* mutants as a percentage of the total count under no-stress (NS) condition. **(C)** Percentage of nuclei in the three states after 24 and 48 h of HS. (n is the total number of analyzed nuclei).

To test if heat-stress duration may cause changes in the chromatin decondensation, which result in the transposition frequency of *ONSEN* subjected to HS, we investigated the heterochromatin condensation status of *drd1*, *ddr*, *nrpd1*, and the wild type under NS and HS conditions by staining the nuclei with DAPI. We classified heterochromatin into the following three states: condensed, half dispersed, and dispersed (**Figure 4A**). The condensed state of heterochromatin accounted for about 66–82% of the total heterochromatin in all plants (**Figure 4B**), and the nuclei of all plants exhibited different degrees of condensation by HS, but the degree of condensation in the nuclei did not differ significantly after 24 and 48 h of HS (**Figure 4C**). After 24 and 48 h of HS, approximately 56% and 73% of the nuclei in WT were decondensed, respectively, whereas, in the *drd1*, *ddr*, and *nrpd1* mutants, the degree of decondensation was not higher than that exhibited by the wild type. HS leads to chromatin decondensation, but the degree of decondensation does not positively correlate with the number of *ONSEN* transpositions. Our results suggest that the decondensation of heterochromatin may facilitate the transcriptional activation of *ONSEN*, but it does not directly affect *ONSEN* transposition.

### 3.6 DNA hypomethylation affects *ONSEN* transcript levels but is not directly related to the transposition

**Figure 5:**
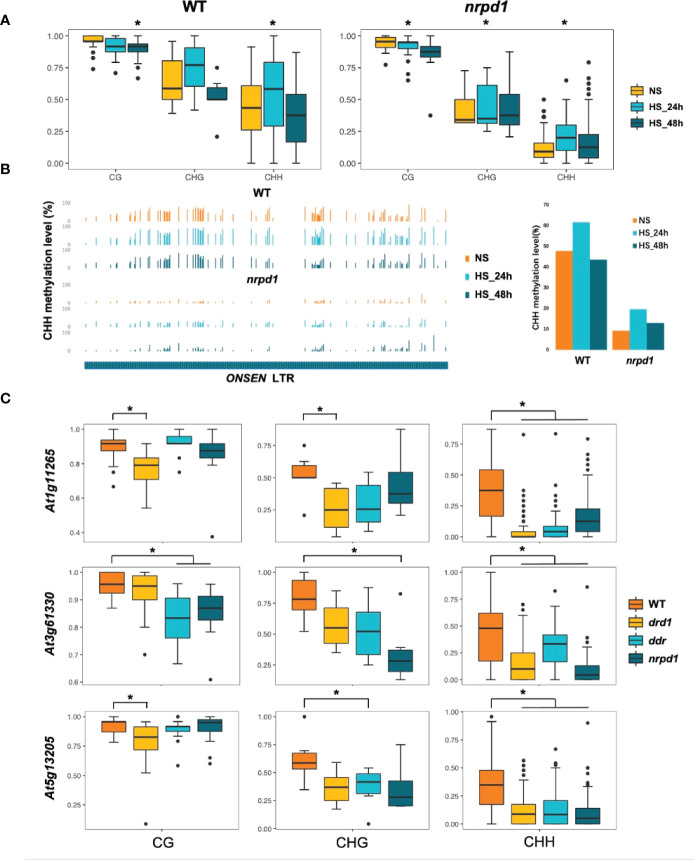
Relationship between duration of high-temperature treatment and *ONSEN* DNA methylation levels. **(A)** Boxplots show the CG, CHG, and CHH (H = A, T, C) methylation levels of *ONSEN* (*At1g11265*) in wild type and *nrpd1* mutant. Asterisks indicate significant differences compared to NS (Student’s *t*-test, P < 0.05). **(B)** CHH methylation levels of the LTR region of *ONSEN* (*At1g11265*) in wild type and *nrpd1* mutant under non-stress (NS) and heat-stress (24 and 48 h). **(C)** DNA methylation levels (CG, CHG, and CHH (H = A, T, C)) of *ONSEN* (*At1g11265*, *At3g61330*, and *At5g13205*) in wild type and *drd1*, *ddr*, and *nrpd1* mutants after 48 h of heat stress. Asterisks indicate significant differences compared to WT (Student’s *t*-test, P < 0.05).

To understand whether silencing of *ONSEN* transposition in the DDR mutants is associated with DNA methylation, we investigated DNA methylation levels in plants exhibiting *ONSEN* transposition under NS and HS (48 h) conditions. The methylation levels of the three copies of *ONSEN* in the *drd1*, *drd1/dms3*, *ddr*, and *nrpd1* mutants, i.e., *At1g11265*, *At3g6133*, and *At5g61305*, were analyzed by Chop-PCR. *drd1*, *drd1/dms3*, and *ddr* mutants exhibited the same DNA methylation levels as that of the *nrpd1* mutant (**Supplementary Figure 5**). However, DNA methylation levels of *ONSEN* in all mutants after 48 h of HS were higher than those under NS condition (**Supplementary Figure 5**). Next, we analyzed the DNA methylation levels of *At1g11265* in the *nrpd1* mutant and the wild type under NS and HS conditions (24 and 48 h) using bs-seq. After HS, the CG methylation level in the *At1g11265* region was slightly decreased in the *nrpd1* mutant and the wild type compared to that under NS condition (**Figure 5A**). Interestingly, except for the CHG methylation level in WT, the methylation of CHG and CHH increased significantly after 24 h of HS and gradually decreased to the same level as under NS conditions after 48 h (**Figure 5A**). This implies that the methylation levels of *ONSEN* may be in dynamic equilibrium during HS. A significant decrease was observed in the level of CHH methylation in the LTR region of *ONSEN* in the *nrpd*1 mutant compared to that in the wild type (**Figure 5B**). However, in both wild type and *nrpd1* mutant, the level of CHH methylation of *ONSEN* was independent of heat treatment (**Figure 5B**).

The Chop-PCR method can only approximate the degree of methylation (**Supplementary Figure 5**). Therefore, to accurately determine the difference in the CG, CHG, and CHH methylation levels of the *ONSEN* region in the wild type and the *drd1*, *ddr*, and *nrpd1* mutants, we investigated the DNA methylation levels of *At1g11265*, *At3g6133*, and *At5g13205* after 48 h of HS using bs-seq. The CHH methylation levels of three copies of *ONSEN* were significantly reduced in *drd1*, *ddr*, and *nrpd1* compared to the wild type (**Figure 5C**). the CG methylation levels of *At1g11265* and *At5g13205* were strongly lost in the DRD1 deletion mutation, but the CG methylation levels of *At3g6133* were significantly reduced in *ddr* and *nrpd1* (**Figure 5C**). The rate of loss of CHG methylation levels also differed across mutants in different *ONSEN* copies compared to WT, but all showed a slight trend of loss (**Figure 5C**). Previous studies have shown that the activity of each copy is different (Cavrak et al., 2014). The difference in methylation levels across copies may be responsible for the different activities. In conclusion, the results suggest that the deletion of *drd1* and *ddr* results in impaired methylation of the *ONSEN* region, which may be one of the reasons for the extensive upregulation of *ONSEN* in the *drd1* and *ddr* mutants. However, the frequency of *ONSEN* transpositions in the *drd1* and *ddr* mutants is lesser than that in the *nrpd1* mutant, implying that the impairment of DNA methylation of *ONSEN* does not directly lead to *ONSEN* transposition.

## 4 Discussion

Our results indicate that the level of DNA methylation in the *ONSEN* region is reduced in DDR mutants, which is consistent with the overall DNA methylation level. This result implies that the absence of the DDR complex may result in impaired function of Pol V, resulting in lower DNA methylation levels. These results are consistent with those of previous reports (Kanno et al., 2004; Kanno et al., 2008; Sasaki et al., 2014). Since the DNA methylation levels of HS plants subjected to 48 h did not change significantly compared to NS levels (**Figure 5A, B**), and *ONSEN* transposition occurred after 48 h of HS, we investigated the methylation levels of mutants in which *ONSEN* underwent transposition after 48 h of HS. The deletion of *drd1* and *ddr* resulted in the loss of DNA methylation in the *ONSEN* region, particularly the methylation of CHH (**Figure 5**). These data suggest that the DDR complex is essential in mediating the DNA methylation pathway for the *ONSEN*. Previous studies have shown that *ONSEN* transcript levels increase in non-CG methylation-impaired mutants (*cmt2, cmt2/3*, and *drm1/2*) under HS condition (Nozawa et al., 2021). This is consistent with our findings that *ONSEN* transcript levels increase in DDR mutants that impaired non-CG methylation.

Our results elucidated that the copy number of *ONSEN* was significantly increased under HS condition in the DDR complex-deficient plants (**Figures 3A, B**). However, a higher copy number does not imply more transposition, as we observed *ONSEN* transposition only in the *ddr* mutants after 48 h of HS (**Figures 3C, D**). The number of *ONSEN* transposition in *ddr* mutants was significantly lower than that in *nrpd1* mutants, although no differences were observed in the transcript levels and copy number of *ONSEN* (**Figures 2**, **3**). This result implies that RdDM-related factors may play different roles in the transcriptional and transpositional repression of *ONSEN*. The loss of the DDR complex releases *ONSEN* transposition silencing (**Figure 3D**). However, among the mutants of DRD1, DMS3, and RDM1, *ONSEN* transposition was observed only in the *drd1* after 48 h of HS (**Figure 3E**). As Wongpalee et al. (2019) reported that DMS3 and RDM1 do not induce the RdDM pathway in DRD1 and PolV mutants (*nrpe1*), it is likely that deletion of DMS3 and RDM1 alone in the present study would only result in reduced repression of *ONSEN* transcription and not transposition (**Figure 3A**). These results suggest that DRD1 may play a key role in the DDR complex-dependent repression of *ONSEN* transposition. *ONSEN* transposition frequencies between DRD1 single mutants and multiple mutants containing DRD1 need to be analyzed in a large number of samples to allow for statistical analysis.

In the RdDM pathway, DRD1 not only forms DDR complexes with DR complexes but also interacts with Pol V (Wierzbicki et al., 2008; Law et al., 2010; Wongpalee et al., 2019). However, interactions of DMS3 and RDM1 with Pol V have not been reported (Gao et al., 2010; Law et al., 2010). This suggests that DRD1 may be a bridge between the DR complex and Pol V (Wongpalee et al., 2019). Hayashi et al. (2020) reported that the transposition of *ONSEN* was observed in the *nrpe1* mutant, a loss-of-function mutant of Pol V. Law et al. (2010) suggested that DRD1 could help initiate or elongate intergenic noncoding (IGN) by remodeling chromatin. This suggests that deletion of *drd1* may result in a partial loss of function of Pol V, resulting in the release of *ONSEN* transposition. Among the components of DDR, *ONSEN* transposition was observed only in mutants deficient in DRD1. The observation supports the function of DRD1that interact with Pol V. However, the exact mechanism behind how the deletion of *drd1* impairs Pol V function is unclear.

**Figure 6:**
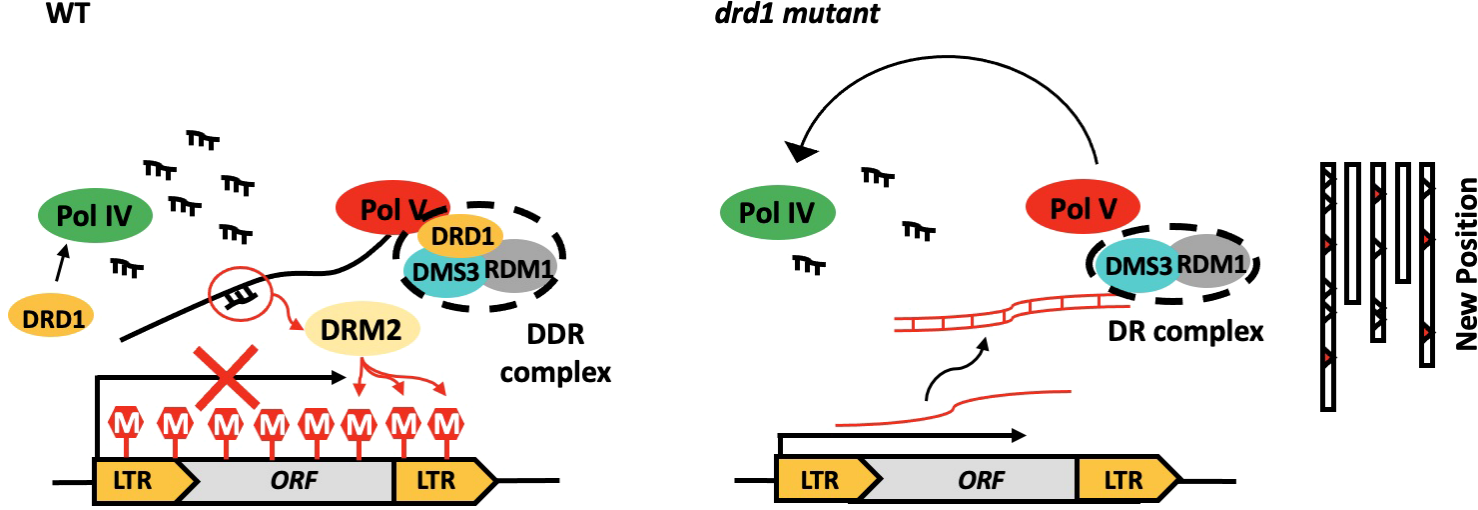
Hypothetical model of silencing of *ONSEN* by DDR complexes. In the wild type, Pol IV and PolV produce siRNA and lncRNA, respectively, resulting in high methylation levels of the *ONSEN*. High DNA methylation levels result in the silencing of *ONSEN* transcription. In addition, no *ONSEN* transposition occurred in WT. In *drd1*, the deletion of DRD1 prevents the DR complex from binding to Pol V. It may also affect the localization of NRPD1b. This leads to a partial loss of function of Pol IV and Pol V, which indirectly affects the amount of siRNA and lncRNA accumulation *in vivo*, leading to the deregulation of *ONSEN* transcription and transposition silencing pathway.

The frequency of *ONSEN* transposition is related to the duration of HS (**Figure 2**). The transgenerational *ONSEN* transposition was detected after 48 h of HS in *ddr* mutant (**Figures 3C–E**). Consistent with what we reported previously (Takehira et al., 2021), *ONSEN* transposition in *drd1* did not occur after 24 h of HS (**Supplementary Figure 4**). These results suggest that HS for a sufficient duration is required for the release of *ONSEN* transposition *via* DRD1-mediated pathway. Wierzbicki et al. (2008) suggested that transposon silencing requires a combination of Pol V transcription and siRNA production. DRD1 affects the localization of NRPD1b (NRPE1), which may indirectly affect siRNA abundance (Pontes et al., 2006). However, siRNA abundance is reduced in the *nrpd1b (nrpe1)* mutant, but it is not entirely eliminated (Pontes et al., 2006). Therefore, we propose a hypothesis that the deletion of *drd1* may impair the function of both Pol V and Pol IV, indirectly leading to a decrease in lncRNA and siRNA abundance, thus impairing the completion of the RdDM process (**Figure 6**). However, since lncRNA and siRNA are not completely eliminated, extended periods of environmental stress are required to activate *ONSEN*. Future research on the role of lncRNAs and siRNAs in the silencing mechanism of *ONSEN* is required.

DAPI can deeply stain the centromere portion of heterochromatic regions. Since the centromeric heterochromatin was already relaxed after 24 hours of heat treatment, we could not see any difference in the degree of relaxation between this treatment and 48 hours of heat treatment. However, we cannot rule out the possibility that *ONSEN* insertion occurred since the chromatin state may be more relaxed at 48 hours than at 24 hours, a degree that cannot be detected in the DAPI staining experiments. It has been previously reported that in the heat-treated progeny of *nrpd1* mutants, more than 80% of the new insertion sites for *ONSEN* are in the gene (Ito et al., 2016). It has been reported that *ONSEN* tends to transpose specific chromatin states, especially regions rich in H2A.Z histones and H3K27me3 histone marks (Roquis et al., 2021). This suggests that mutants of the DDR complex may alter particular chromatin states. Future analysis of *ONSEN* transposition target preference will be of interest for studying the transpositional regulation pathways. In conclusion, the DDR complex contributes to silencing *ONSEN*. These results highlight the role of RdDM factors in regulating *ONSEN* activity. Thus, it is essential to discuss each regulatory mechanism separately when the transcriptional and transpositional regulatory mechanisms of retrotransposons are being discussed. We also provide evidence that the intensity of environmental stress leads to the loss of the transposon silencing mechanism.

## Data availability statement

The original contributions presented in the study are included in the article/**Supplementary Material**. Further inquiries can be directed to the corresponding author.

## Author contributions

XN and LC coordinated this work. XN performed cytological experiments. XN, AK and HI wrote the paper. All authors have read and agreed to the submission of the manuscript. All authors contributed to the article and approved the submitted version.
