# Supplementary material for: Regulatory mechanism of a heat-activated retrotransposon by DDR complex in Arabidopsis thaliana
Source: Front Plant Sci. 2022 Dec 21;13:1048957. doi: 10.3389/fpls.2022.1048957 (PMC9811314; doi:10.3389/fpls.2022.1048957)
Supplement: Supplementary file 1 [file Presentation_1.pdf]

Supplementary Material

1 Supplementary Figures

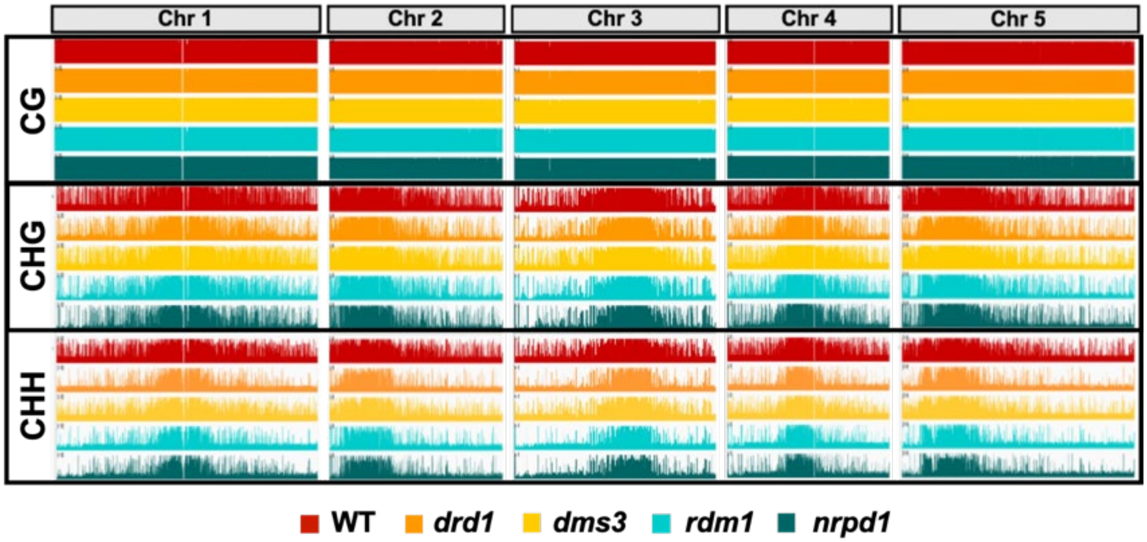

Supplementary Figure 1. Deletion of WT, *DRD1*, *DMS3*, *RDM1,* and *NRPD1* results in reduced DNA methylation levels for the whole genome. Snapshots of genome browsers showing DNA methylation (CG, CHG, CHH (H = A, T, G)) levels in wildtype and *drd1*, *dms3*, *rdm1*, and *nrpd1* mutants.

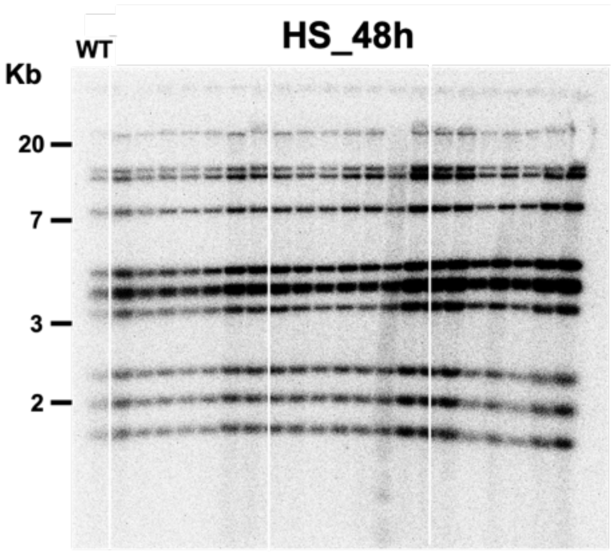

Supplementary Figure 2. Transposition of *ONSEN* was not observed in the wildtype after 48 h of HS. Southern blotting analysis of *ONSEN* in the next generation. The leftmost band in figure shows the wildtype pattern under non-stress condition.

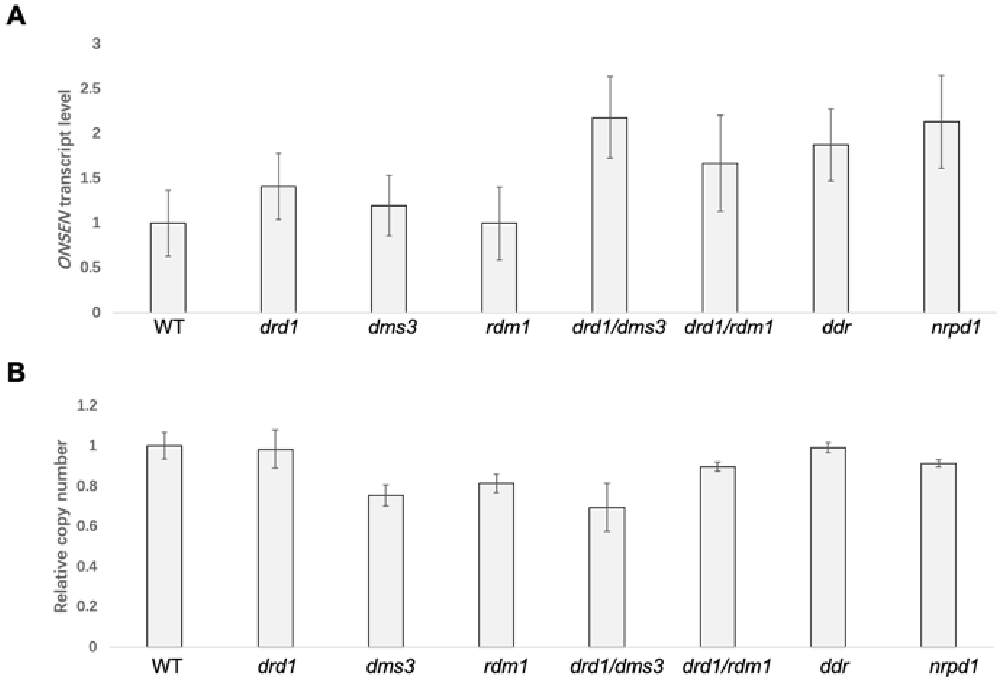

Supplementary Figure 3. Under non-stress conditions, deletion of DDR genes does not activate *ONSEN*. Relative *ONSEN* expression levels (A) and relative *ONSEN* copy number (B) in wildtype and *drd1*, *dms3*, *drd1/dms3*, *drd1/rdm1*, *ddr*, and *nrpd1* mutants under non-stress conditions.

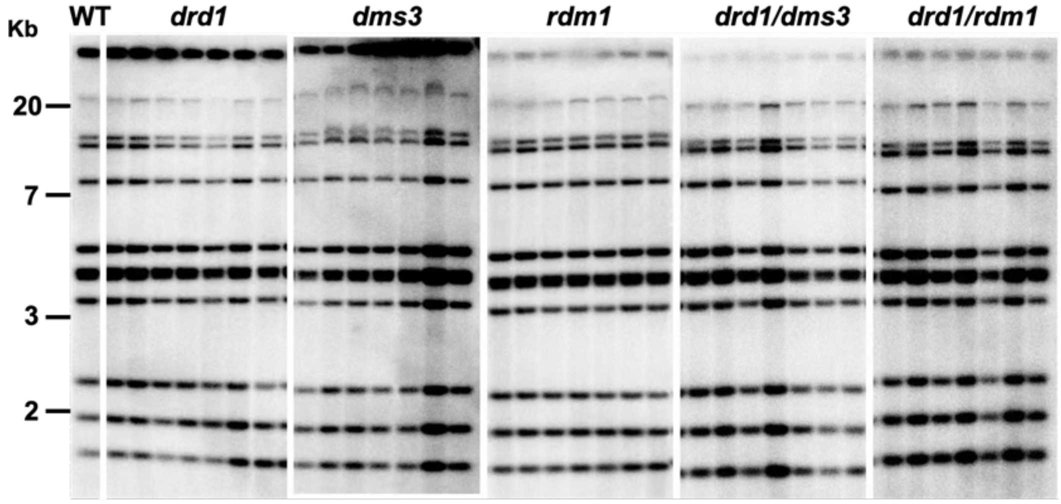

Supplementary Figure 4. *ONSEN* transposition was not observed in the DDR complex-associated mutants after 24 h of heat-stress. Southern blot analysis of *ONSEN* in next-generation *drd1*, *dms3*, *rdm1*, *drd1/dms3*, *drd1/rdm1*, *ddr*, and *nrpd1* mutants. The leftmost band in figure shows the pattern of wildtype under non-stress condition.

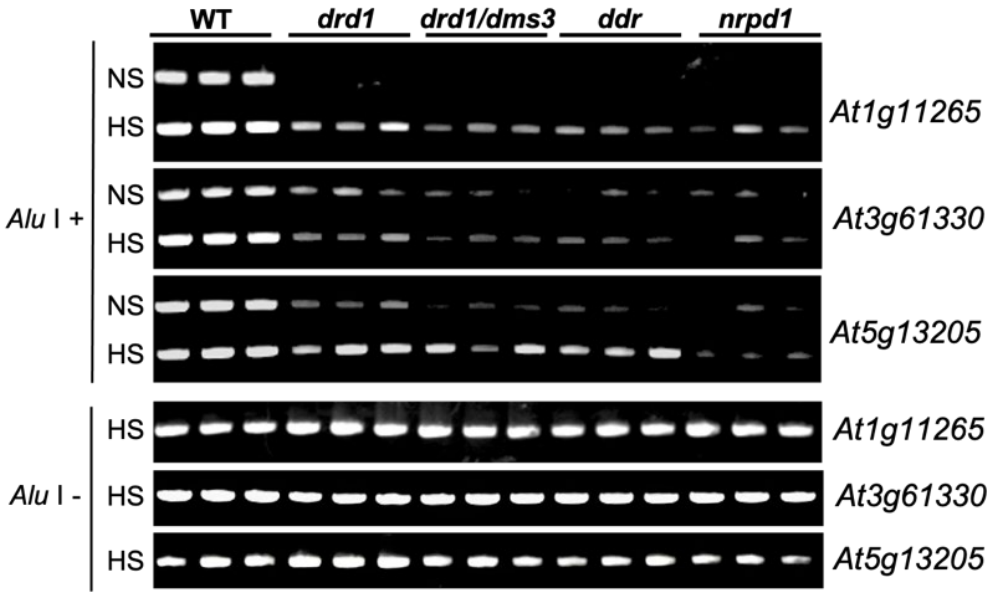

Supplementary Figure 5. *drd1*, *drd1/dms3*, and *ddr* mutants exhibit low DNA methylation levels of the *ONSEN* region. Chop-PCR analysis of the methylation levels of *ONSEN* (*At1g11265*, *At3g61330*, and *At5g13205*) in wildtype and *drd1*, *drd1/dms3*, *ddr* and *nrpd1* mutants after 48 h of heat-stress.
